# Supplementary material for: Bioactive Hydrogel Formulation Based on Ferulic Acid-Grafted Nano-Chitosan and Bacterial Nanocellulose Enriched with Selenium Nanoparticles from Kombucha Fermentation
Source: J Funct Biomater. 2024 Jul 22;15(7):202. doi: 10.3390/jfb15070202 (PMC11277923; doi:10.3390/jfb15070202)
Supplement: Supplementary file 1 [file jfb-15-00202-s001.zip › jfb-3041935-supplementary.pdf]

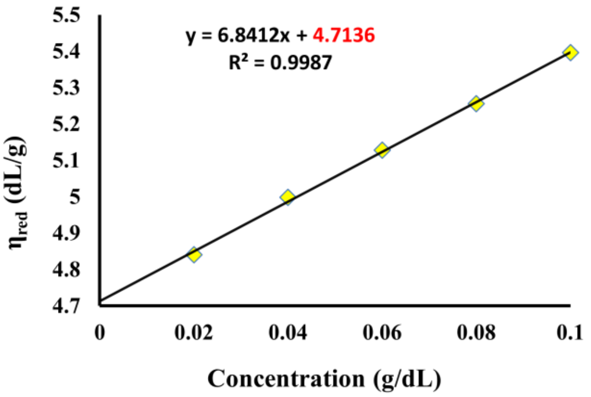

Figure S1. Graphical representation of the reduced viscosity as a function of chitosan (CS) concentration. The intercept, 4.7136 dL/g represents the intrinsic viscosity of CS.

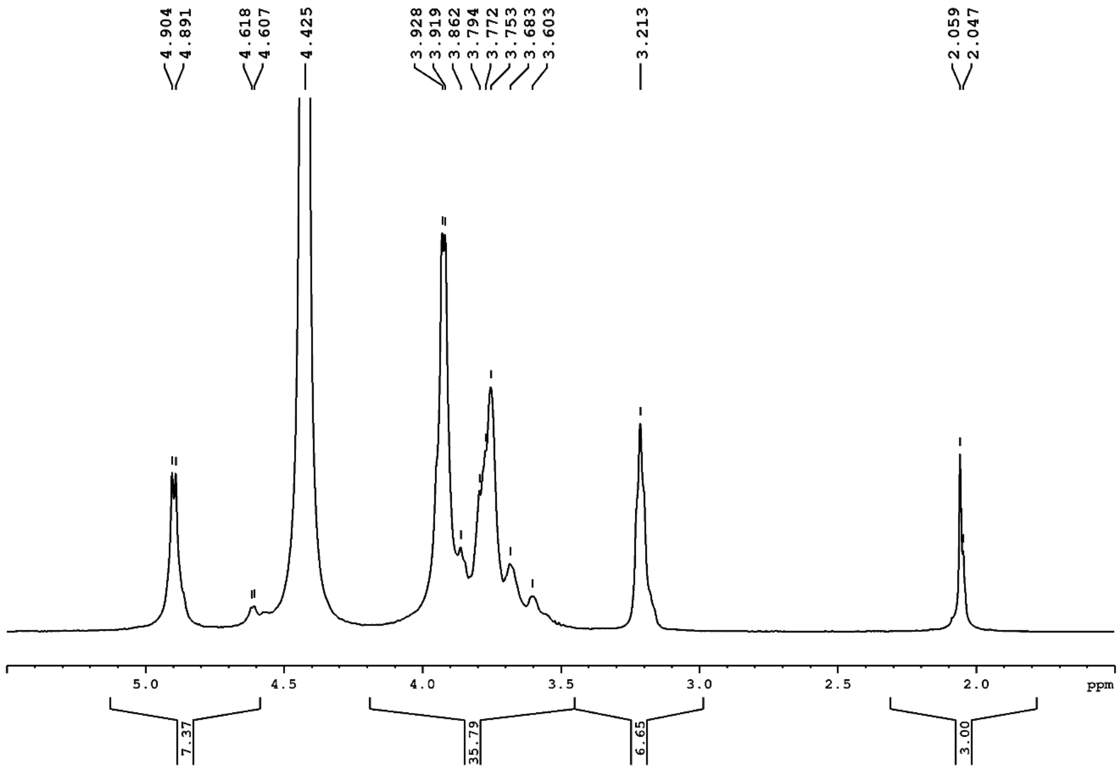

Figure S2. ^{1}H NMR spectrum of fungal chitosan (CS) used in this study

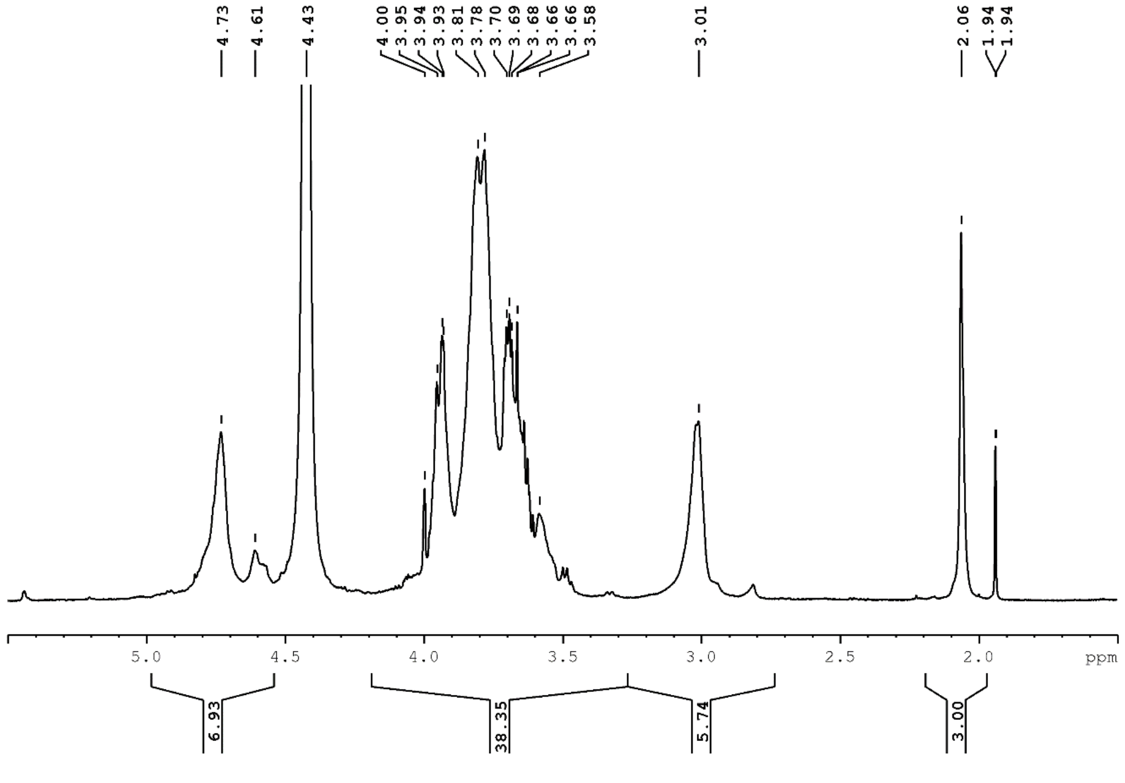

Figure S3. ^{1}H NMR spectrum of CSFa(−), i.e., without addition of ferulic acid in the reaction (non-grafted chitosan)

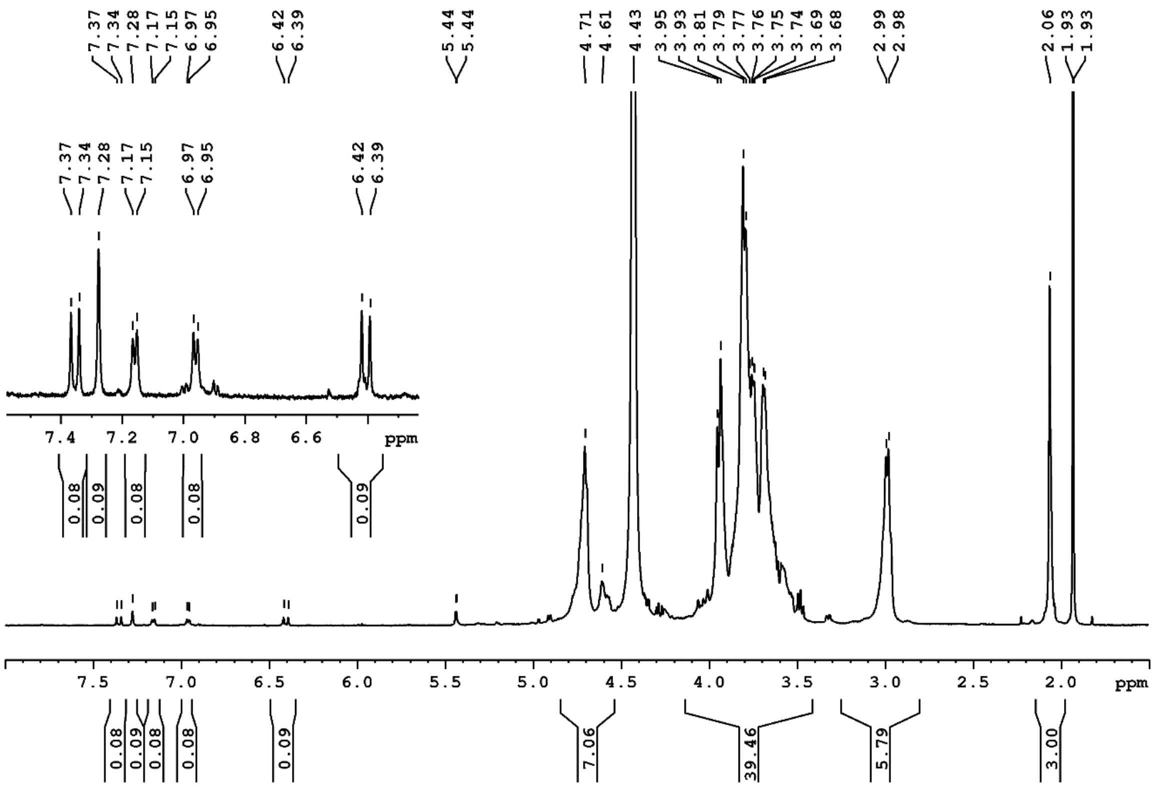

Figure S4. ^{1}H NMR spectrum of CSFa(+), i.e., with addition of ferulic acid in the reaction (grafted chitosan)

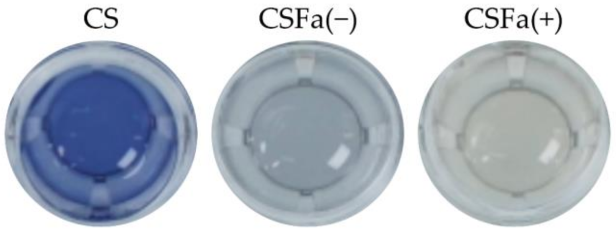

(**a**)

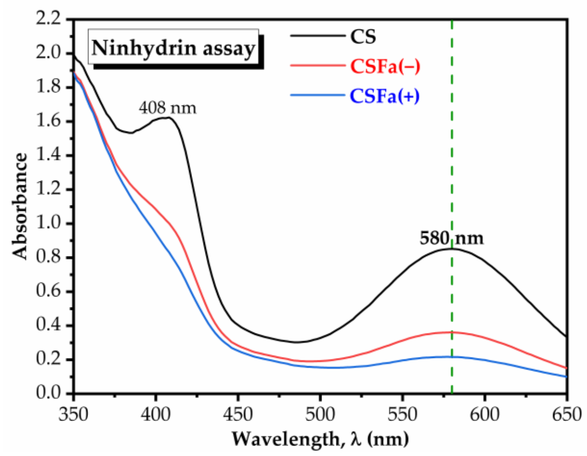

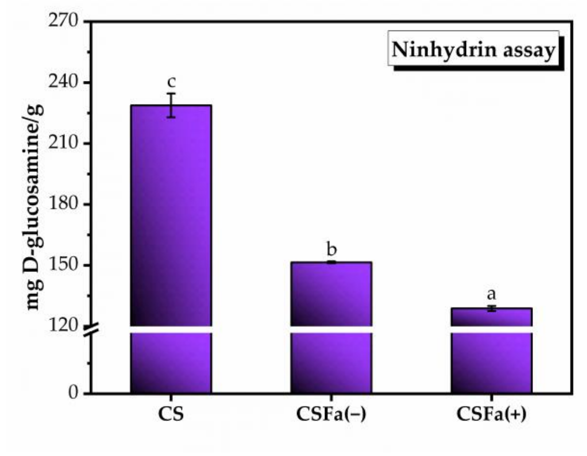

(**b**) (**c**)

Figure S5. Ninhydrin assay: (**a**) The appearance of chitosan solutions following the ninhydrin assay; (**b**) The absorbance spectra of chitosan samples following the ninhydrin assay; (**c**) Quantification of free amino groups by ninhydrin assay (± error bars, α<0.05, n=3, different letters show statistically significant differences between samples); CS: initial chitosan; CSFa(−): non-grafted chitosan, CSFa(+): ferulic acid-grafted chitosan.

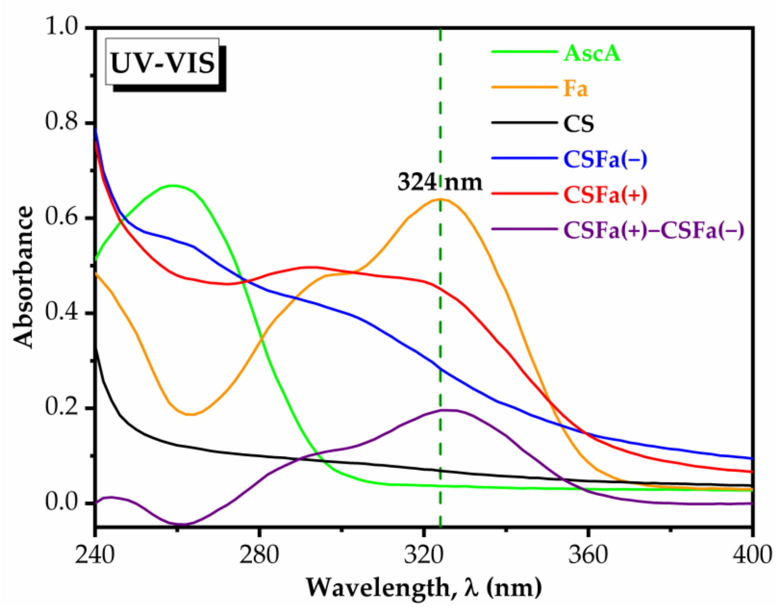

Figure S6. UV-VIS spectroscopy; AscA: ascorbic acid, Fa: ferulic acid, CS: initial chitosan; CSFa(−): non-grafted chitosan, CSFa(+): ferulic acid-grafted chitosan.

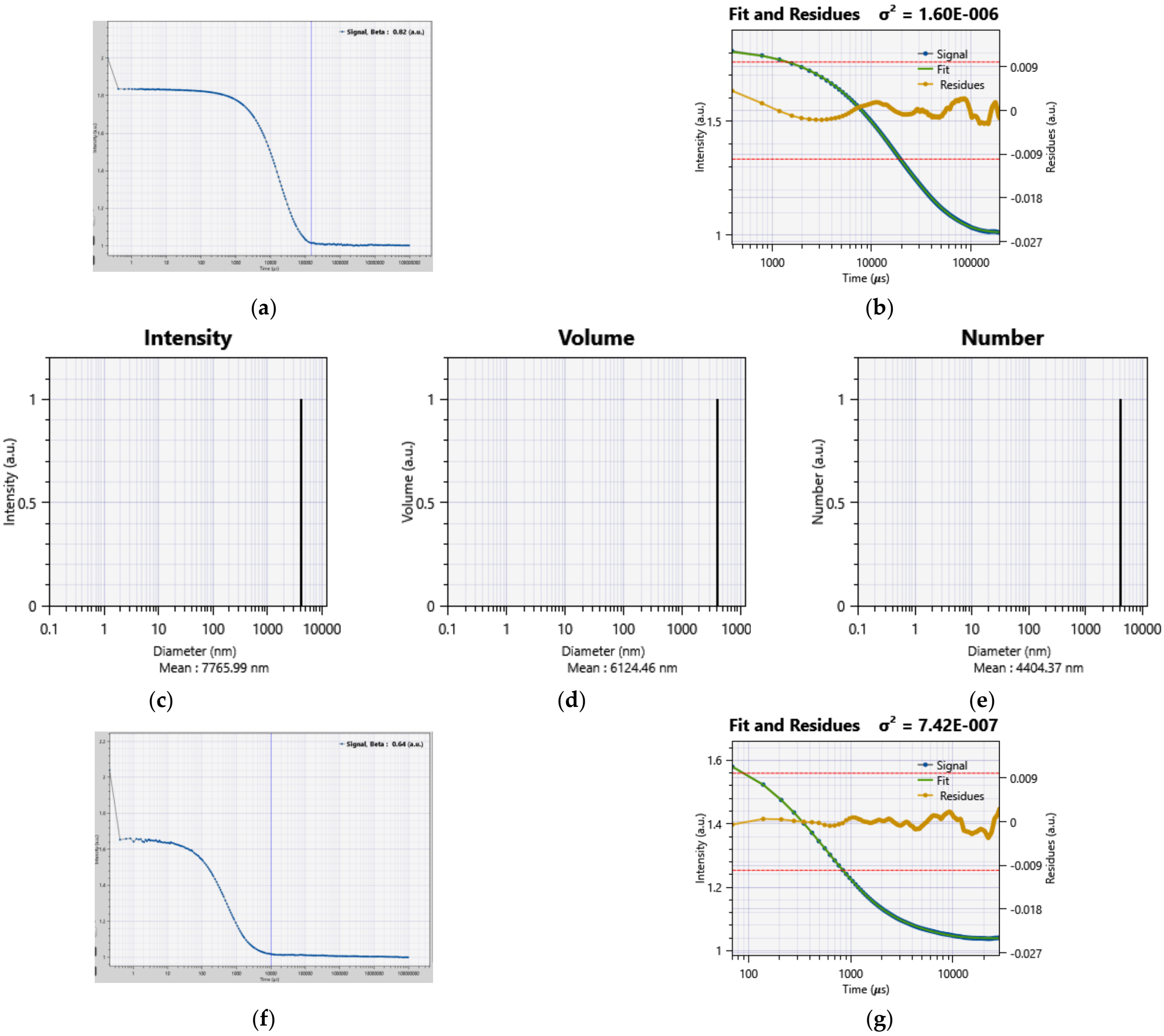

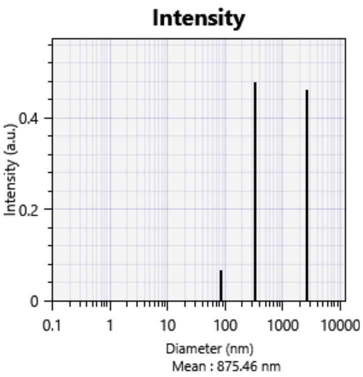

(**h**)

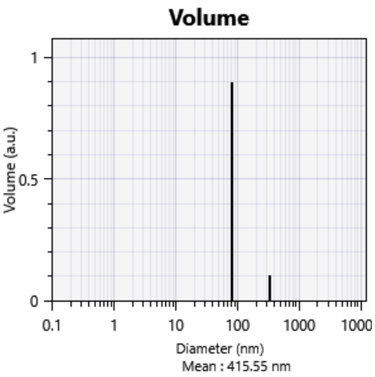

(**i**)

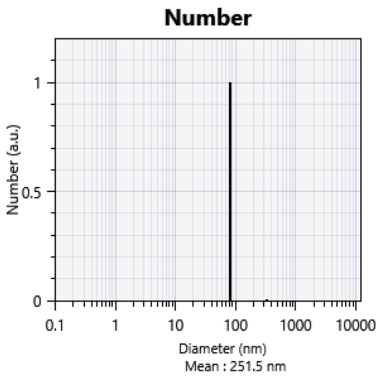

(**j**)

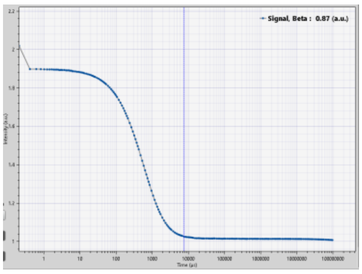

(**k**)

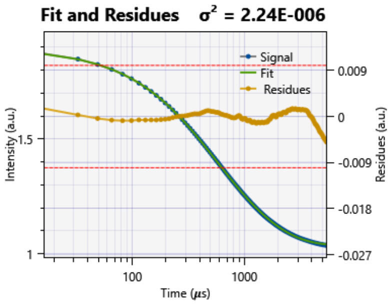

(**l**)

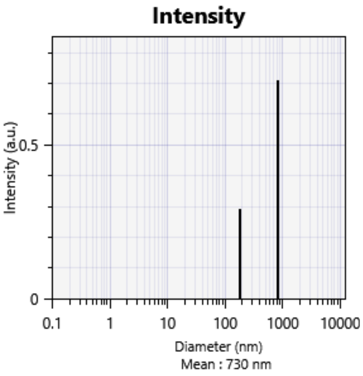

(**m**)

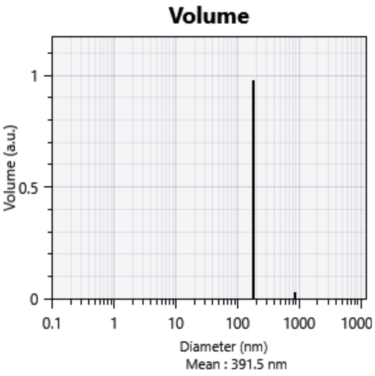

(**n**)

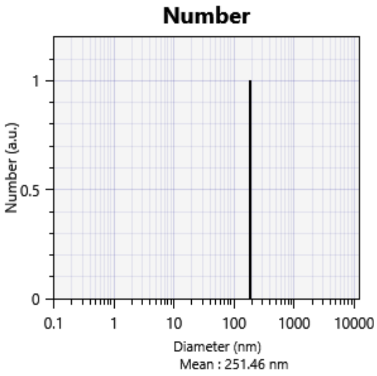

(**o**)

Figure S7. DLS analysis in 2% AcOH using the Pade Laplace (PD) method: (**a**) autocorrelation function for CS; (**b**) simulation of autocorrelation function for CS; (**c**) Intensity for CS; (**d**) Volume for CS; (**e**) Number for CS; (**f**) autocorrelation function for CSFa(−); (**g**) simulation of autocorrelation function for CSFa(−); (**h**) Intensity for CSFa(−); (**i**) Volume for CSFa(−); (**j**) Number for CSFa(−); (**k**) autocorrelation function for CSFa(+); (**l**) simulation of autocorrelation function for CSFa(+); (**m**) Intensity for CSFa(+); (**n**) Volume for CSFa(+); (**o**) Number for CSFa(+); CSFa(−): without ferulic acid addition in the reaction (non-grafted chitosan); CSFa(+): ferulic acid-grafted chitosan.

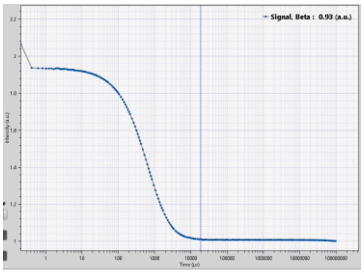

(**a**)

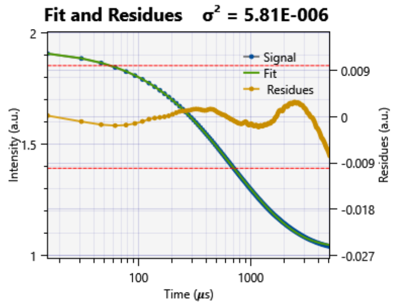

(**b**)

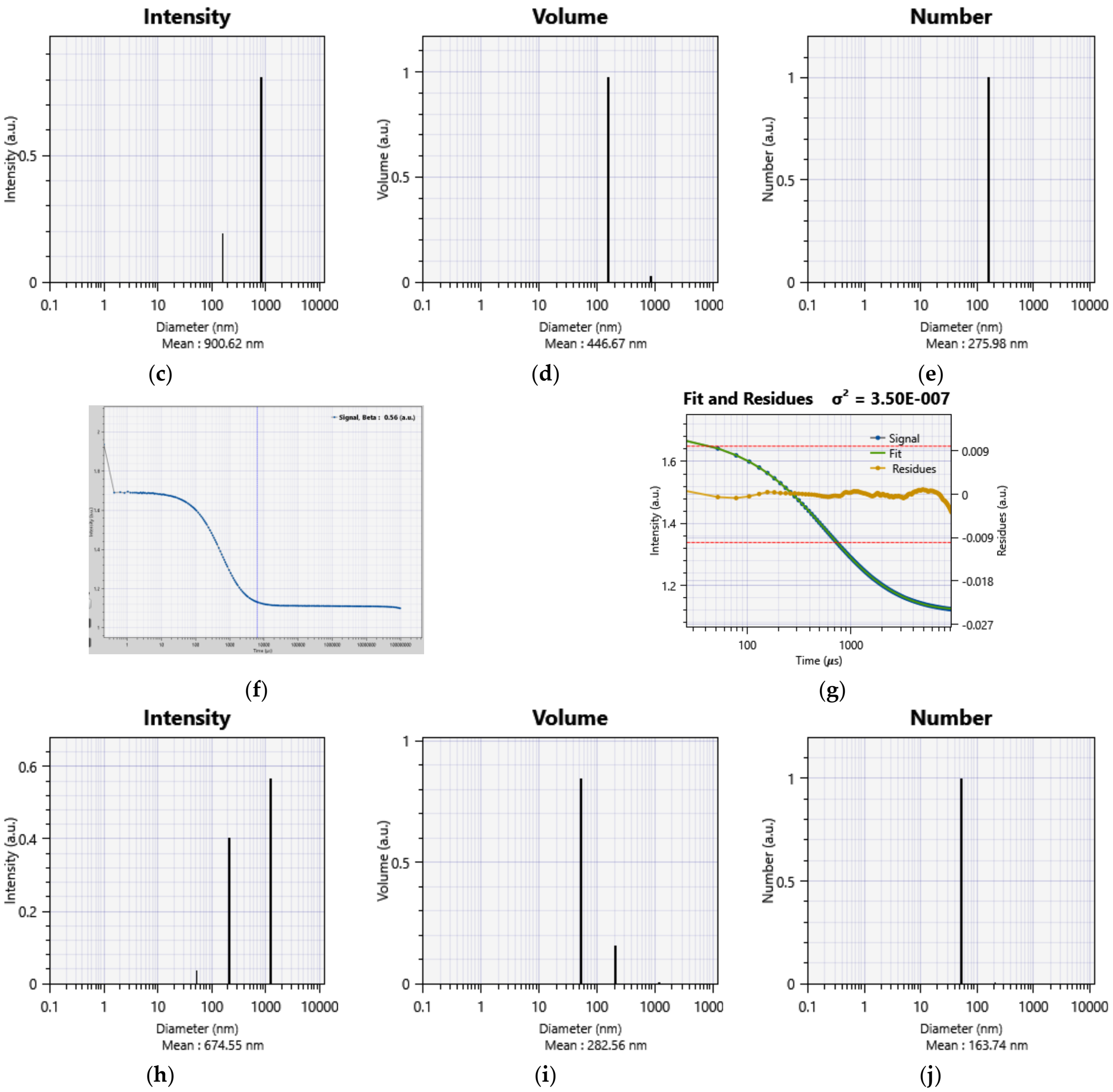

Figure S8. DLS analysis in ddH2O using the Pade Laplace (PD) method: (**a**) autocorrelation function for CSFa(−); (**b**) simulation of autocorrelation function for CSFa(−); (**c**) Intensity for CSFa(−); (**d**) Volume for CSFa(−); (**e**) Number for CSFa(−); (**f**) autocorrelation function for CSFa(+); (**g**) simulation of autocorrelation function for CSFa(+); (**h**) Intensity for CSFa(+); (**i**) Volume for CSFa(+); (**j**) Number for CSFa(+); CSFa(−): without ferulic acid addition in the reaction (non-grafted chitosan); CSFa(+): ferulic acid-grafted chitosan.

Table S1. DLS analysis of CS: initial chitosan; CSFa(−): non-grafted chitosan, CSFa(+): ferulic acid-grafted chitosan in 2% AcOH using the Pade Laplace (PD) method.

Sample	Size (nm)	Intensity (a.u.)	Volume (a.u.)	Number (a.u.)
**CS**	3935.03	0.24	1	1
	18399.11	0.76	-	-
**CSFa(−)**	80.88	0.06	0.9	1
	331.93	0.48	0.1	1.8e^{-3}
	2567.24	0.46	2.0e^{-4}	6.6e^{-9}
**CSFa(+)**	178.69	0.29	0.98	1
	811.29	0.71	0.02	2.6e^{-4}

Table S2. DLS analysis of CSFa(−): non-grafted chitosan, CSFa(+): ferulic acid-grafted chitosan in ddH2O using the Pade Laplace (PD) method.

Sample	Size (nm)	Intensity (a.u.)	Volume (a.u.)	Number (a.u.)

**CSFa(−)**	156.7	0.19	0.97	1
	812.77	0.81	0.03	1.9e-4
**CSFa(+)**	51.67	0.03	0.84	1
	201.02	0.40	0.16	2.9e-3
	1384.76	0.57	1.1e-3	1.1e-7

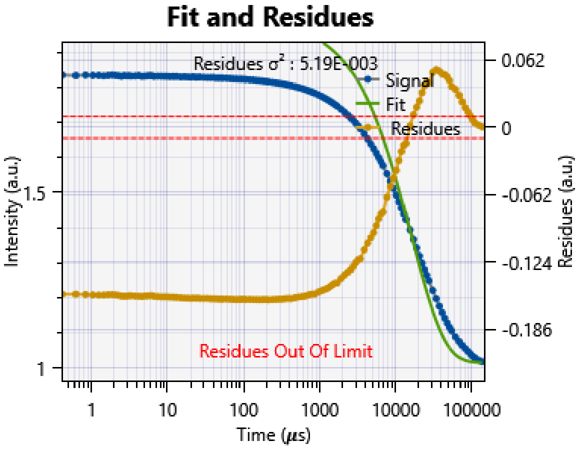

(**a**)

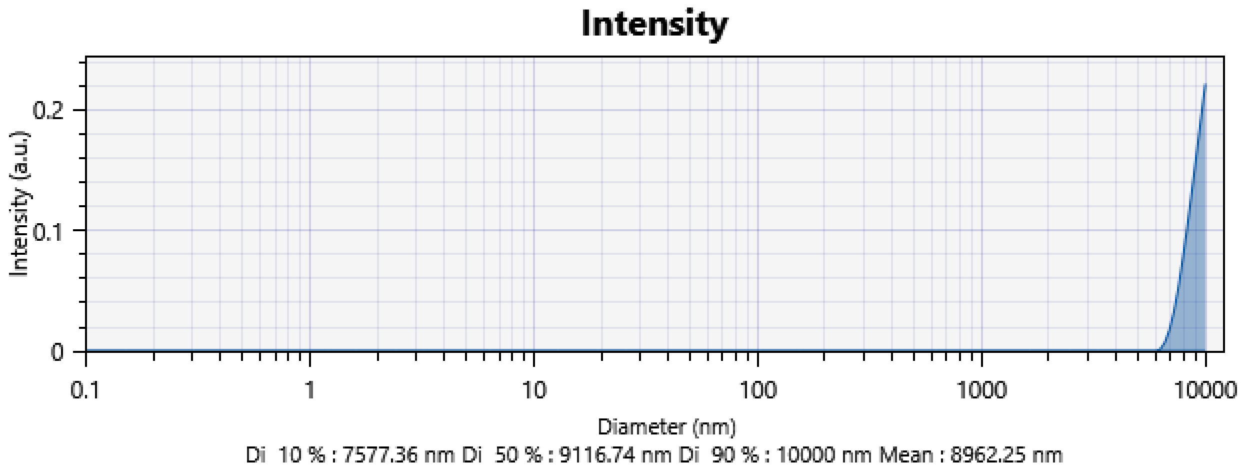

(**b**)

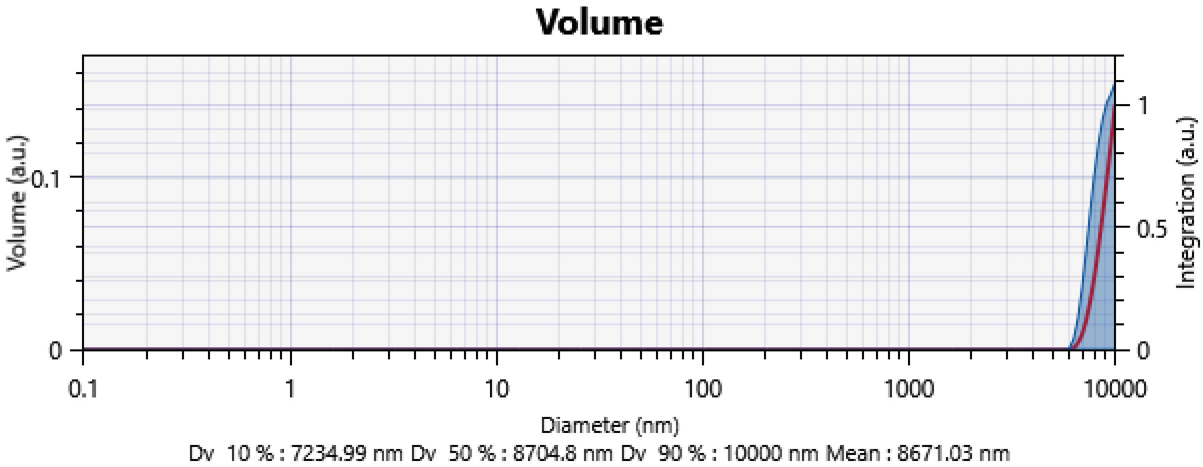

(**c**)

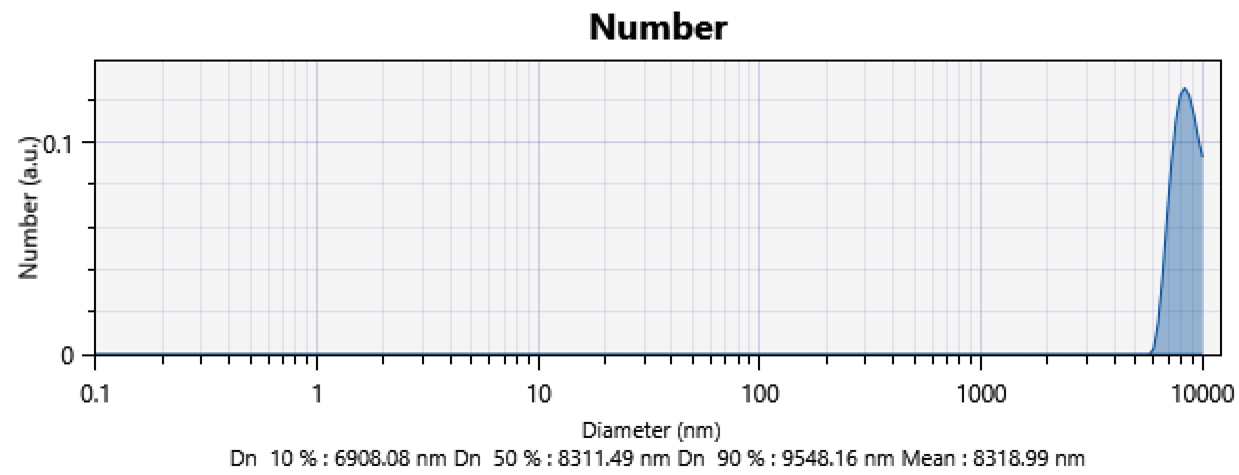
Number
Number (a.u.)
0.1
0
0.1
1
10
100
1000
10000
Diameter (nm)
Dn 10 % : 6908.08 nm Dn 50 % : 8311.49 nm Dn 90 % : 9548.16 nm Mean : 8318.99 nm

(d)

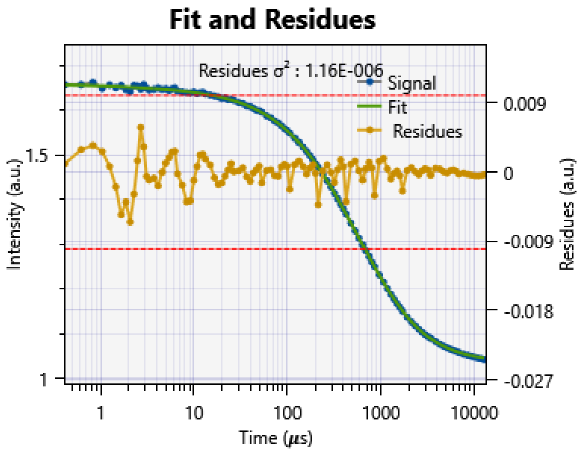
Fit and Residues
Residues σ² : 1.16E-006
Signal
Fit
Residues
Intensity (a.u.)
1.5
1
0.009
0
-0.009
-0.018
-0.027
Residues (a.u.)
1
10
100
1000
10000
Time (μs)

(e)

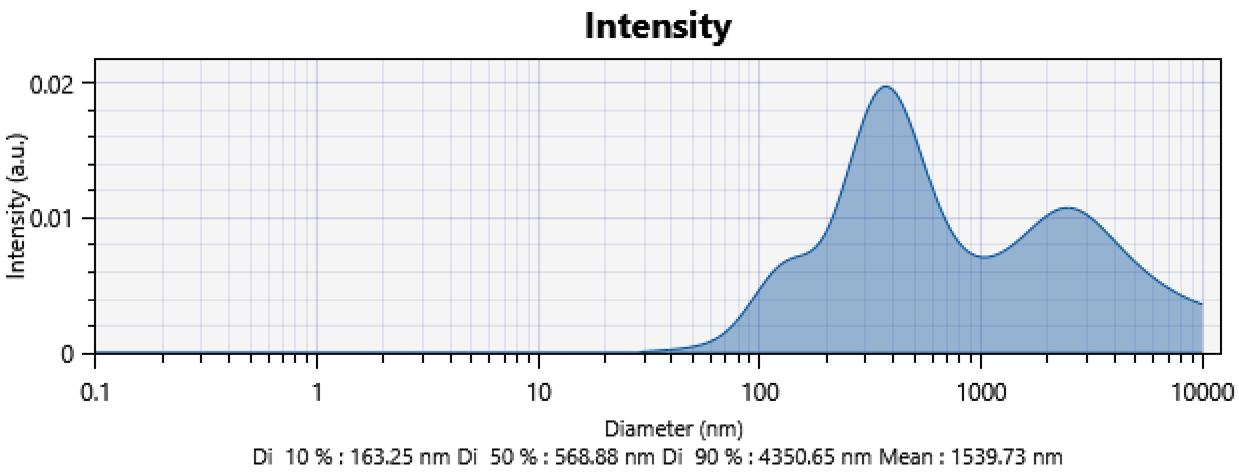
Intensity
Intensity (a.u.)
0.02
0.01
0
0.1
1
10
100
1000
10000
Diameter (nm)
Di 10 % : 163.25 nm Di 50 % : 568.88 nm Di 90 % : 4350.65 nm Mean : 1539.73 nm

(f)

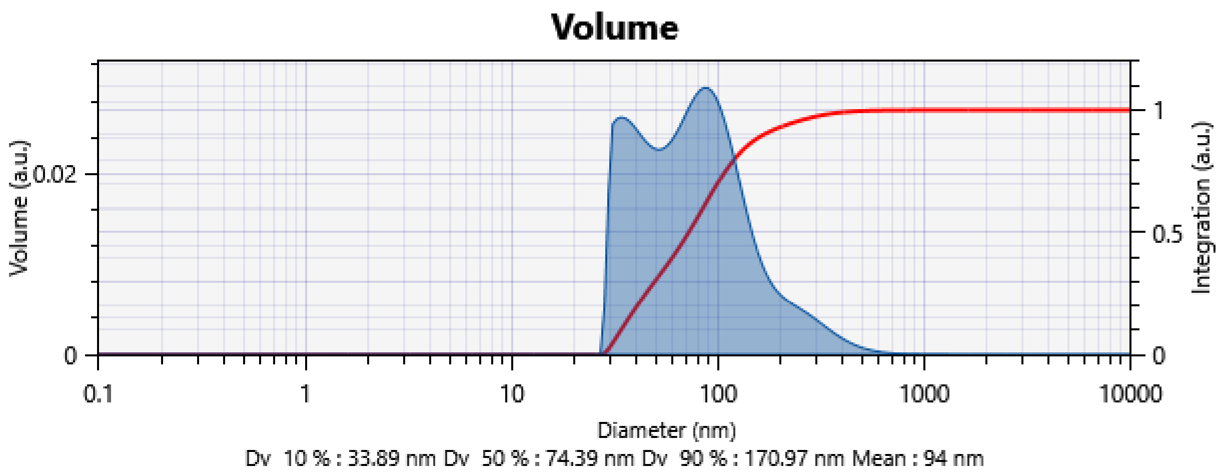
Volume
Volume (a.u.)
Integration (a.u.)
Diameter (nm)
Dv 10 % : 33.89 nm Dv 50 % : 74.39 nm Dv 90 % : 170.97 nm Mean : 94 nm

(g)

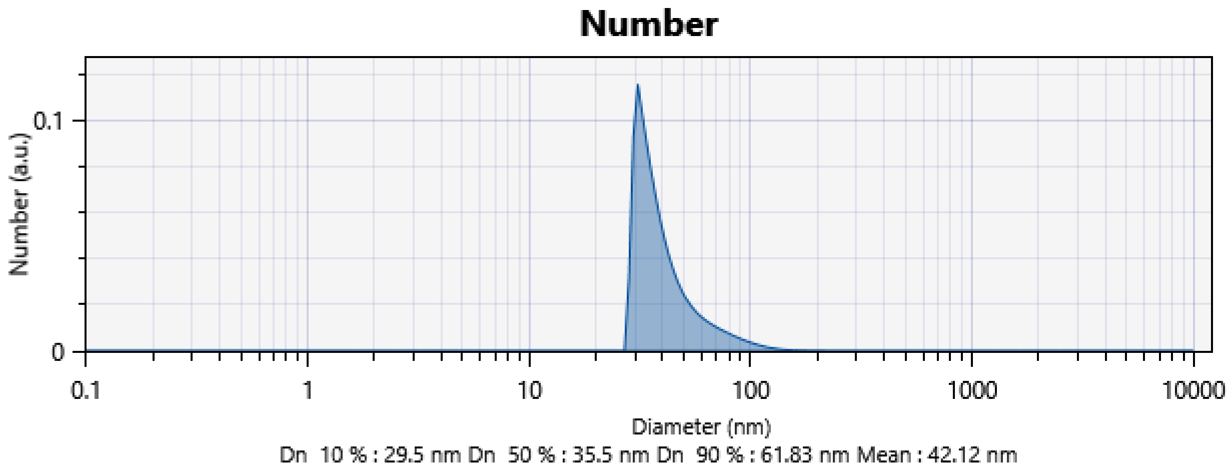
Number
Number (a.u.)
Diameter (nm)
Dn 10 % : 29.5 nm Dn 50 % : 35.5 nm Dn 90 % : 61.83 nm Mean : 42.12 nm

(h)

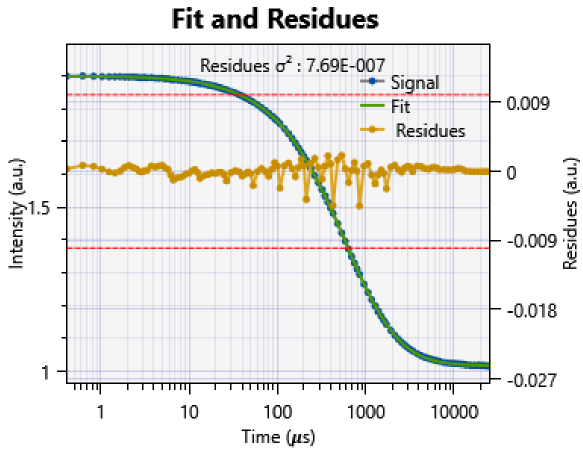
Fit and Residues
Residues σ² : 7.69E-007
Signal
Fit
Residues
Intensity (a.u.)
Residues (a.u.)
Time (μs)

(i)

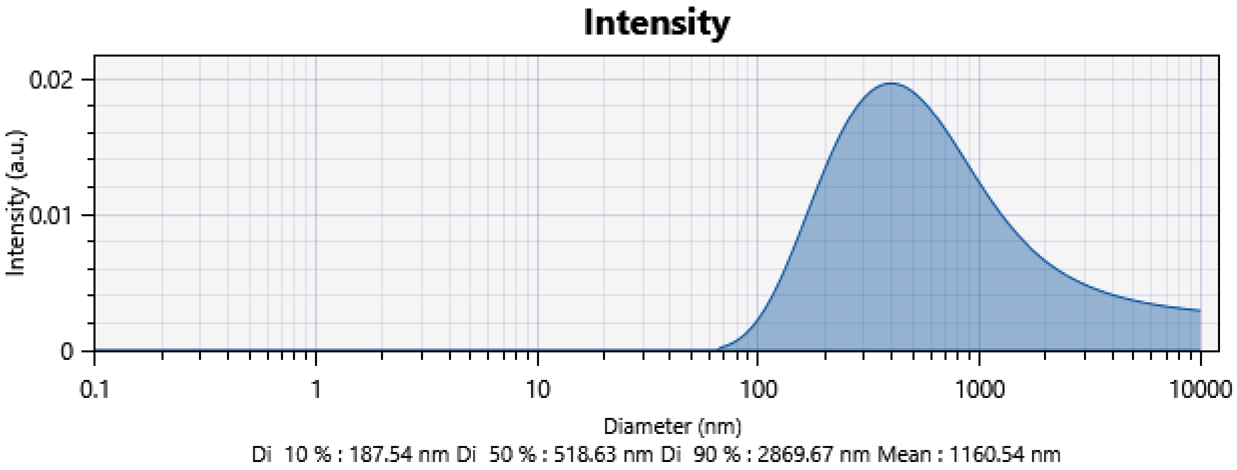

(**j**)

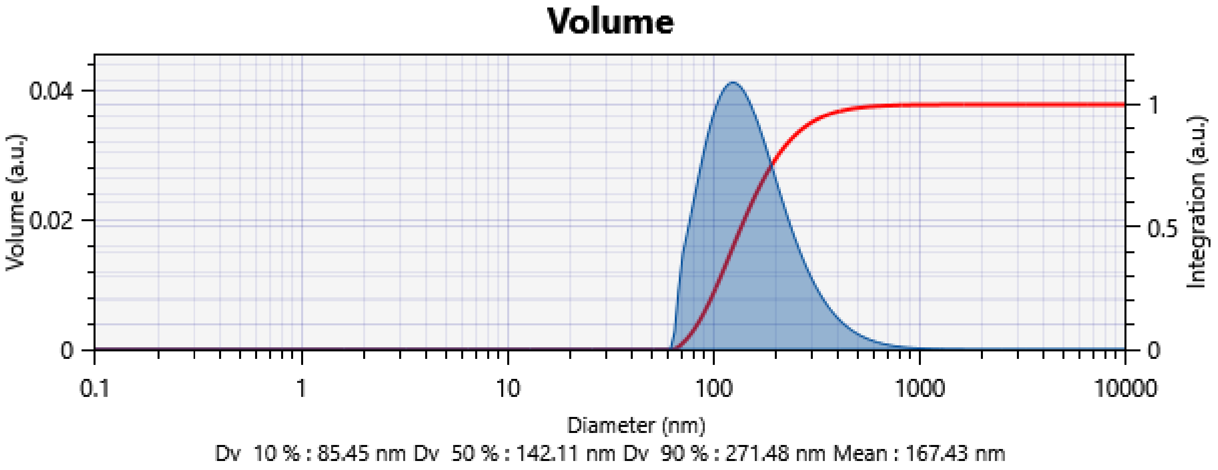

(**k**)

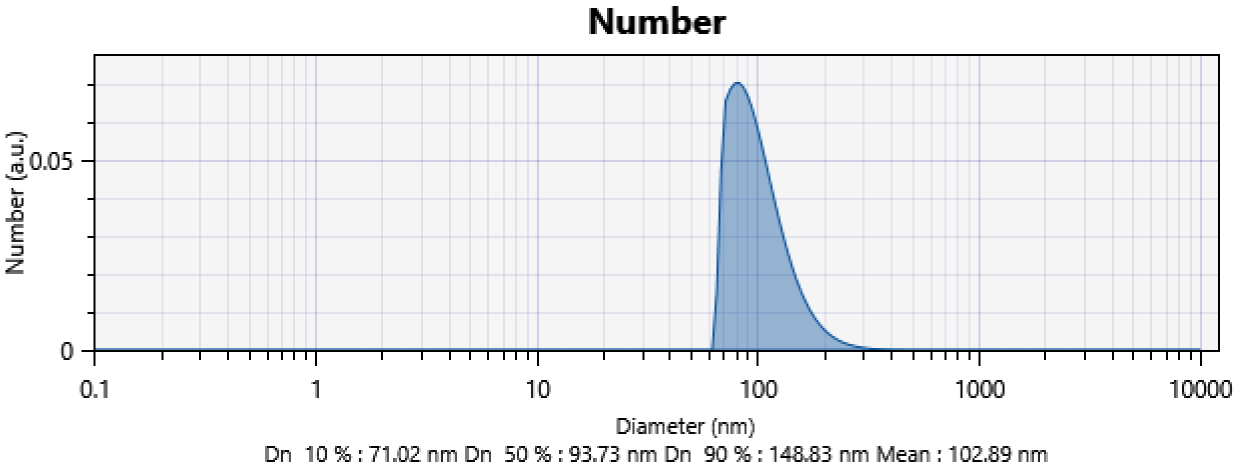

(**l**)

Figure S9. DLS analysis in 2% AcOH using the SBL method: (**a**) simulation of autocorrelation function for CS; (**b**) Intensity for CS; (**c**) Volume for CS; (**d**) Number for CS; (**e**) simulation of autocorrelation function for CSFa(−); (**f**) Intensity for CSFa(−); (**g**) Volume for CSFa(−); (**h**) Number for CSFa(−); (**i**) simulation of autocorrelation function for CSFa(+); (**j**) Intensity for CSFa(+); (**k**) Volume for CSFa(+); (**l**) Number for CSFa(+); CSFa(−): without ferulic acid addition in the reaction (non-grafted chitosan); CSFa(+): ferulic acid-grafted chitosan.

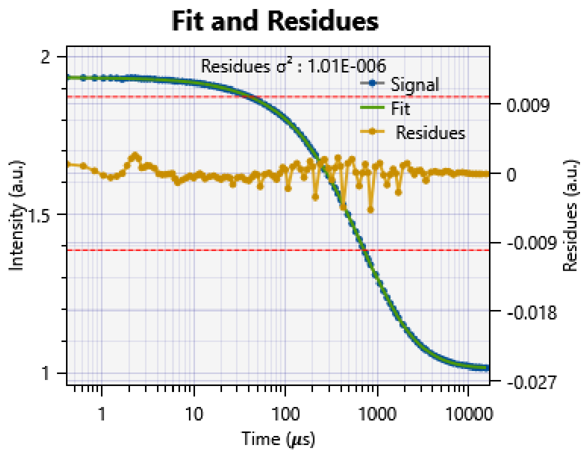
Fit and Residues
Residues σ² : 1.01E-006
Signal
Fit
Residues
Intensity (a.u.)
Residues (a.u.)
2
1.5
1
0.009
0
-0.009
-0.018
-0.027
1
10
100
1000
10000
Time (µs)

(**a**)

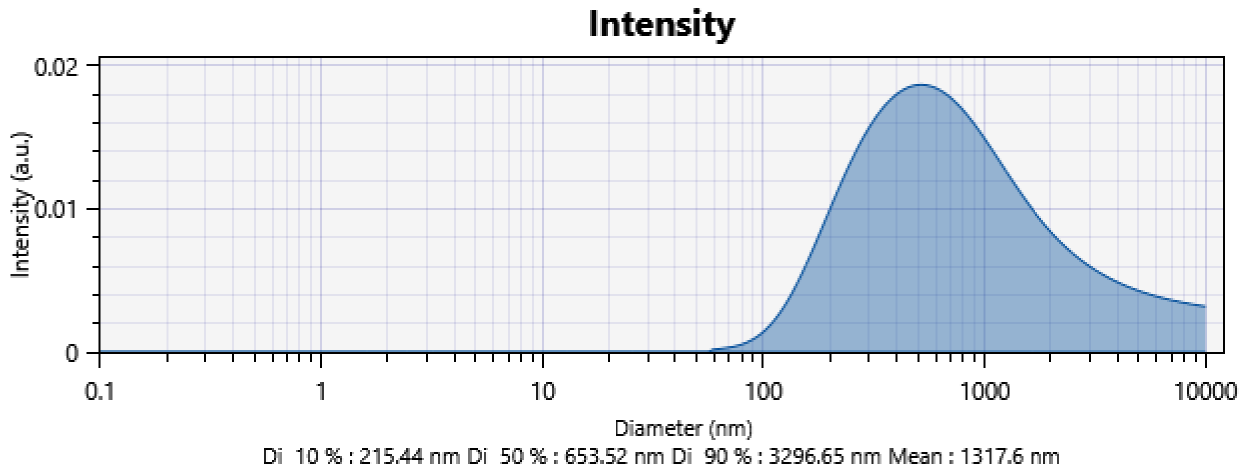
Intensity
Intensity (a.u.)
0.02
0.01
0
0.1
1
10
100
1000
10000
Diameter (nm)
Di 10 % : 215.44 nm Di 50 % : 653.52 nm Di 90 % : 3296.65 nm Mean : 1317.6 nm

(**b**)

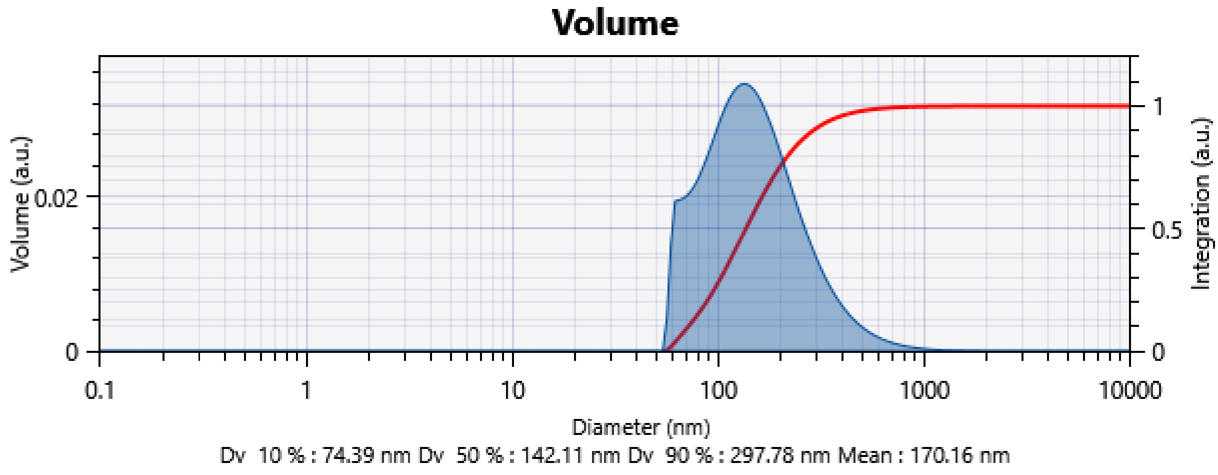
Volume
Volume (a.u.)
Integration (a.u.)
0.02
0
1
0.5
0
0.1
1
10
100
1000
10000
Diameter (nm)
Dv 10 % : 74.39 nm Dv 50 % : 142.11 nm Dv 90 % : 297.78 nm Mean : 170.16 nm

(**c**)

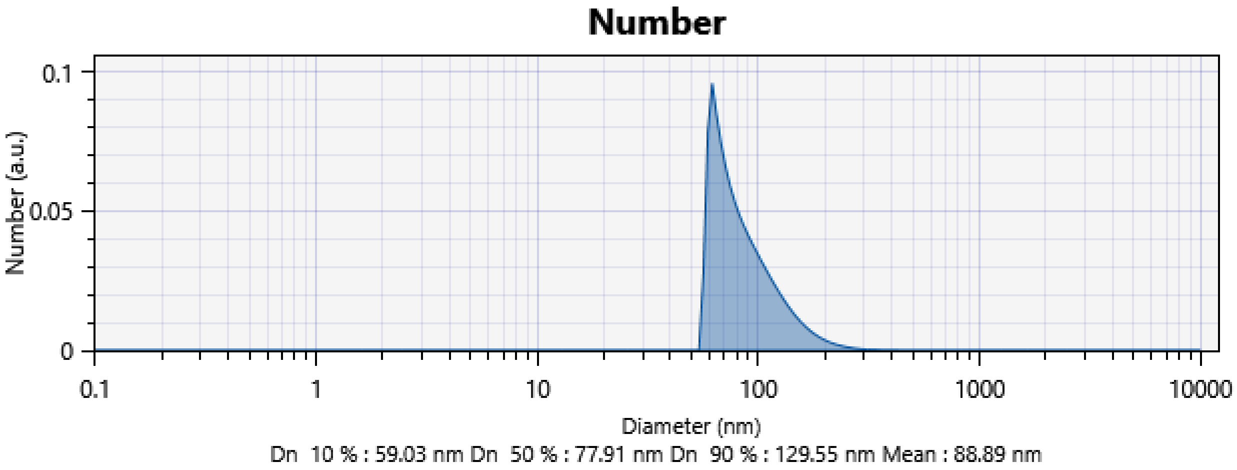
Number
Number (a.u.)
0.1
0.05
0
0.1
1
10
100
1000
10000
Diameter (nm)
Dn 10 % : 59.03 nm Dn 50 % : 77.91 nm Dn 90 % : 129.55 nm Mean : 88.89 nm
(d)

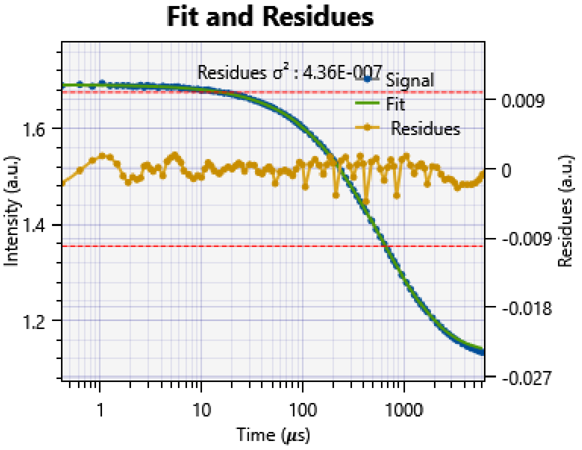
Fit and Residues
Residues σ² : 4.36E-007
Signal
Fit
Residues
Intensity (a.u.)
1.6
1.4
1.2
Residues (a.u.)
0.009
0
-0.009
-0.018
-0.027
1
10
100
1000
Time (μs)
(e)

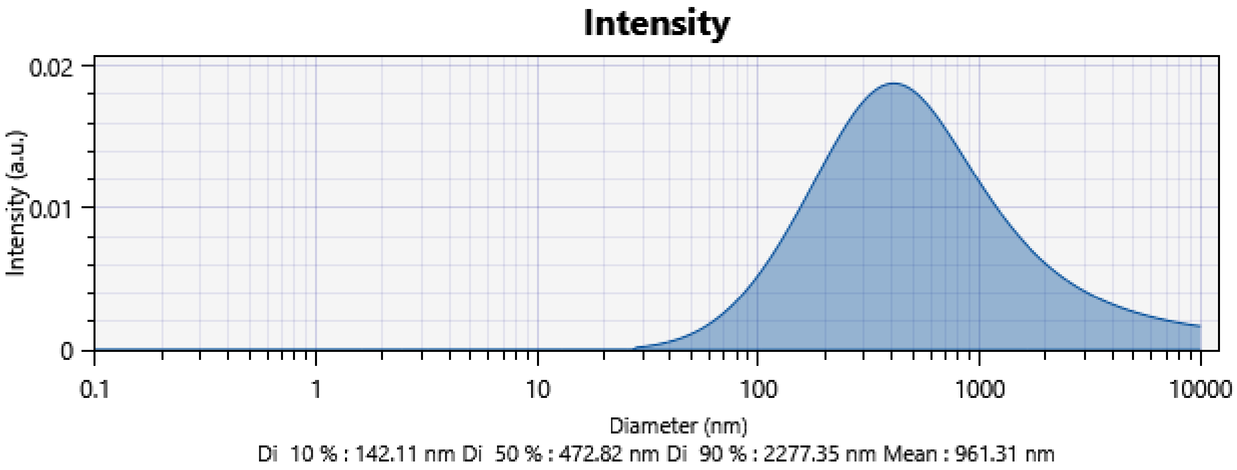
Intensity
Intensity (a.u.)
0.02
0.01
0
0.1
1
10
100
1000
10000
Diameter (nm)
Di 10 % : 142.11 nm Di 50 % : 472.82 nm Di 90 % : 2277.35 nm Mean : 961.31 nm
(f)

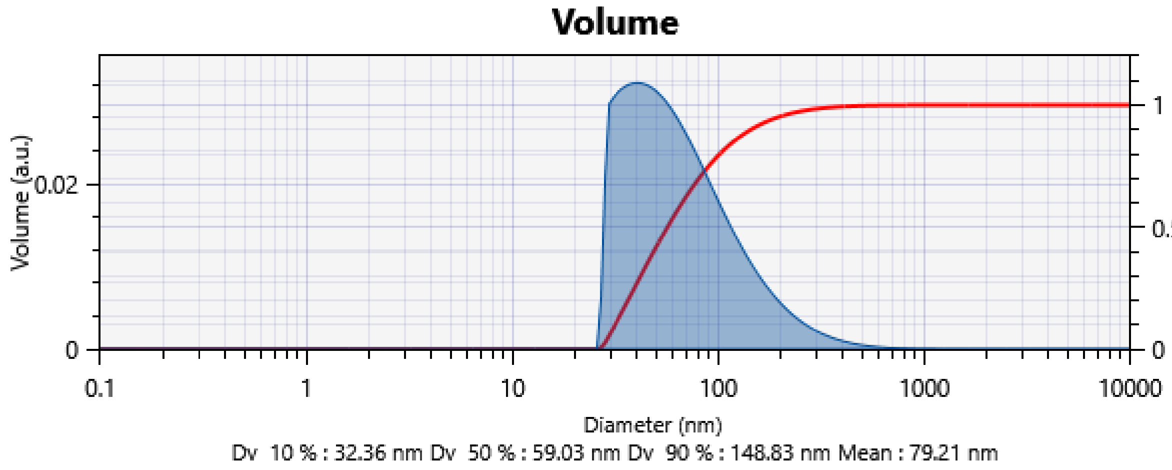

(**g**)

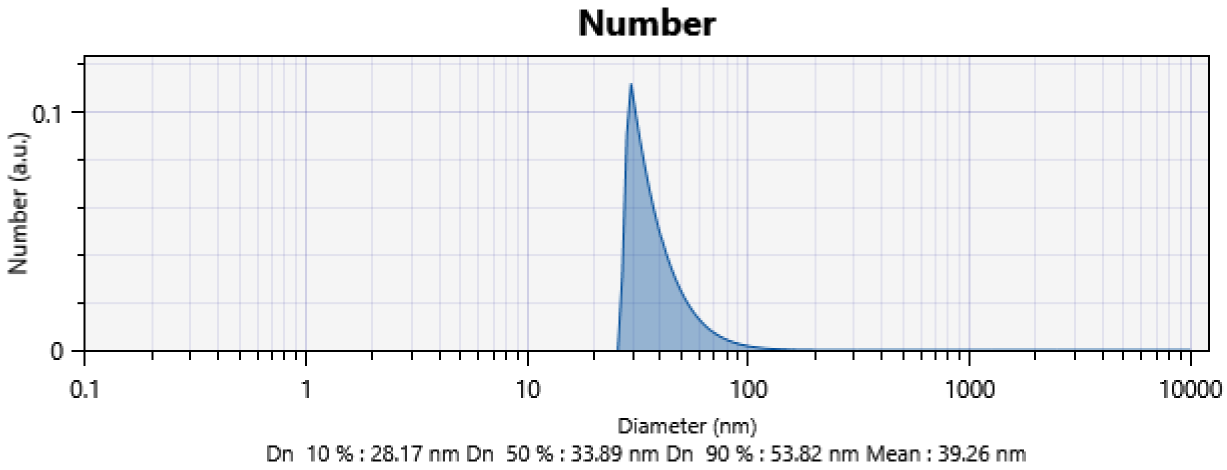

(**h**)

Figure S10. DLS analysis in ddH_2O using the SBL method: (**a**) simulation of autocorrelation function for CSFa(−); (**b**) Intensity for CSFa(−); (**c**) Volume for CSFa(−); (**d**) Number for CSFa(−); (**e**) simulation of autocorrelation function for CSFa(+); (**f**) Intensity for CSFa(+); (**g**) Volume for CSFa(+); (**h**) Number for CSFa(+); CSFa(−): without ferulic acid addition in the reaction (non-grafted chitosan); CSFa(+): ferulic acid-grafted chitosan.

Table S3. DLS analysis of CS: initial chitosan; CSFa(−): non-grafted chitosan, CSFa(+): ferulic acid-grafted chitosan in 2% AcOH using the SBL method.

Sample	Mean size (nm)	Intensity (a.u.)	Volume (a.u.)	Number (a.u.)
**CS**	8519.74	56.86	-	-
	9600.65	43.14	-	-
	8713.04	-	100	-
	7947.98	-	-	65.49
	9416.25	-	-	34.51
**CSFa(−)**	389.9	61.52	-	-
	3444.66	38.48	-	-
	39.22	-	31.23	-
	117.59	-	68.77	-
	42.15	-	-	100
**CSFa(+)**	1184.73	100	-	-
	167.55	-	100	-
	102.96	-	-	100

Table S4. DLS analysis of CSFa(−): non-grafted chitosan, CSFa(+): ferulic acid-grafted chitosan in ddH_2O using the SBL method.

Sample	Mean size (nm)	Intensity (a.u.)	Volume (a.u.)	Number (a.u.)
	1343.55	100	-	-
**CSFa(−)**	170.28	-	100	-
	88.95	-	-	100
	976.02	100	-	-
**CSFa(+)**	79.26	-	100	-
	39.23	-	-	100

Table S5. Polydispersity Index, PDI (a.u.)

Solvent	CS	CSFa(−)	CSFa(+)
**2% AcOH**	0.34	0.30	0.24
**ddH₂O**	-	0.28	0.36

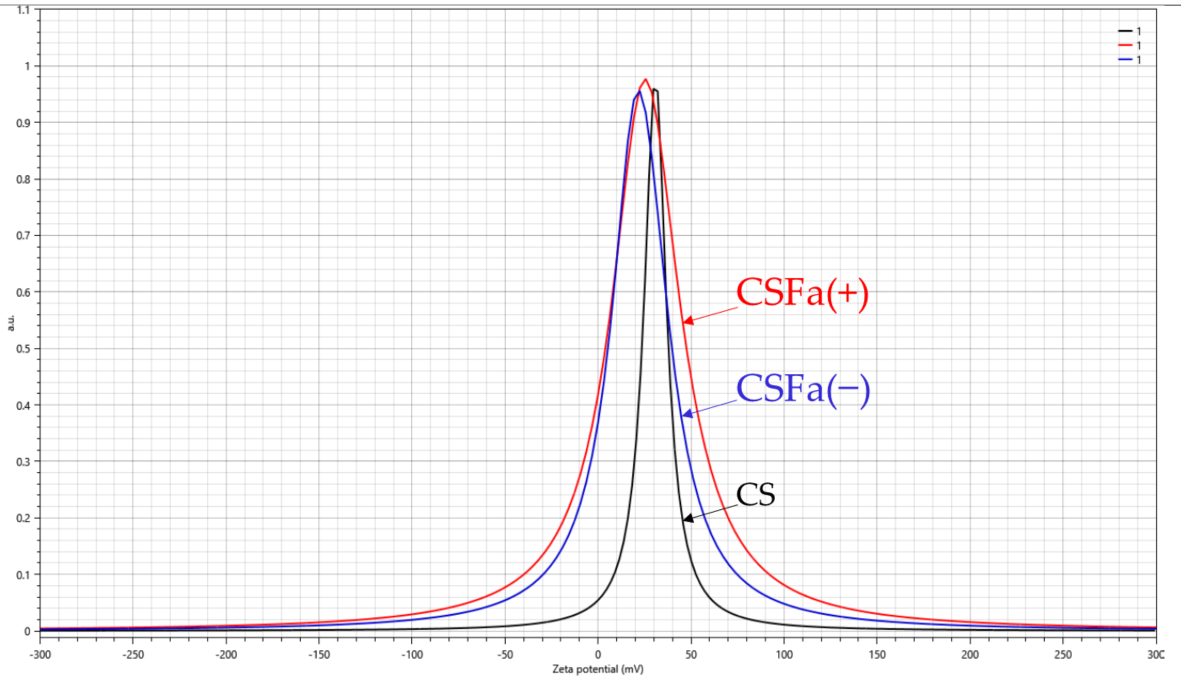

(**a**)

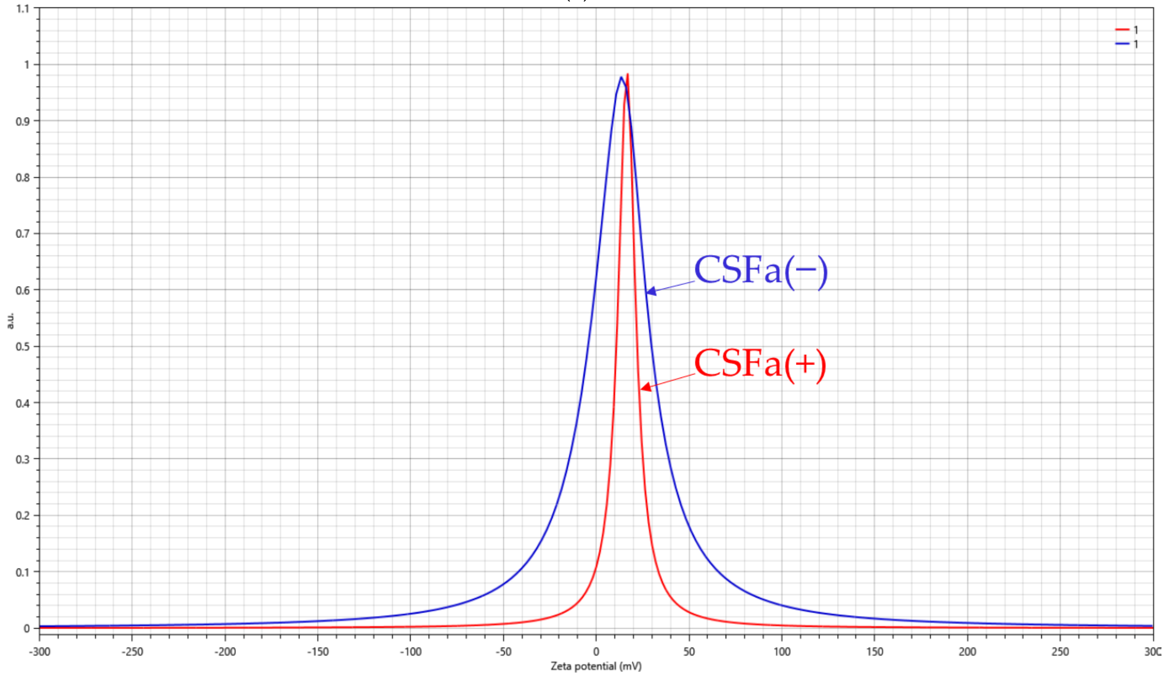

(**b**)

Figure S11. Zeta potential: (**a**) in 2% acetic acid (AcOH); (**b**) in double-distilled water (ddH2O); CS: initial chitosan; CSFa(−): non-grafted chitosan; CSFa(+): ferulic acid-grafted chitosan.

Table S6. Zeta potential, ζ (mV) ± standard deviation (*n*=6) of CS: initial chitosan; CSFa(−): non-grafted chitosan, CSFa(+): ferulic acid-grafted chitosan.

Solvent	CS	CSFa(−)	CSFa(+)
**2% AcOH**	30.66 ± 1.12	22.38 ± 3.54	25.69 ± 3.91
**ddH2O**	-	12.66 ± 2.95	16.38 ± 0.61

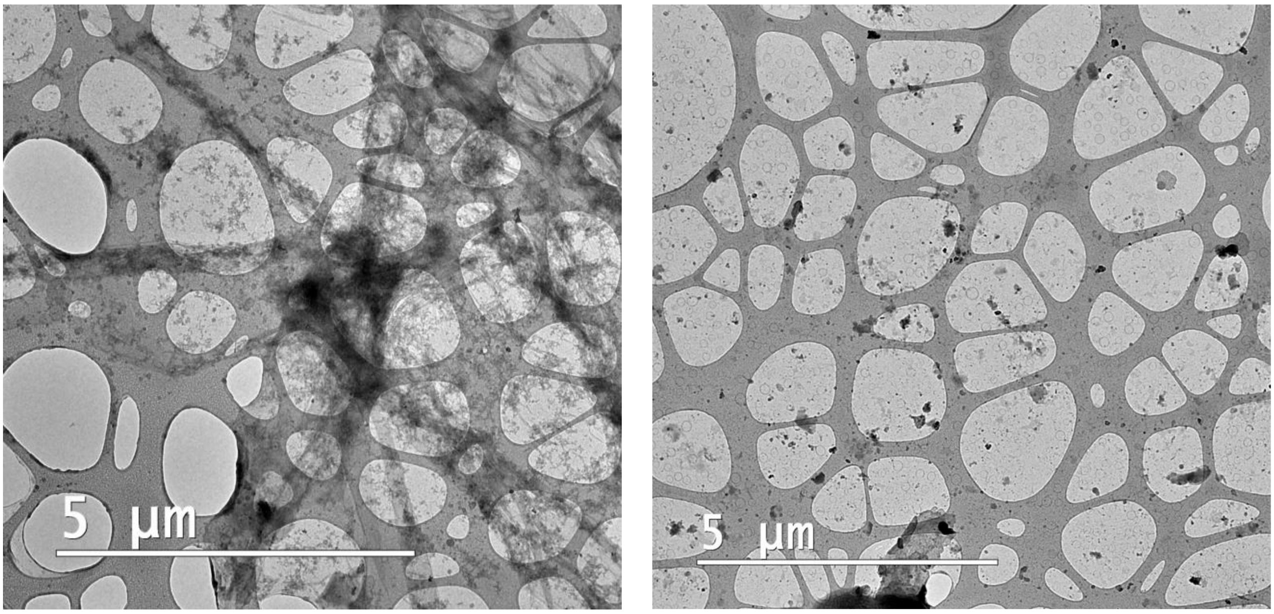

(**a**) (**b**)

Figure S12. TEM analysis: (**a**) CSFa(−), i.e., without ferulic acid addition in the reaction (non-grafted chitosan); (**b**) CSFa(+), i.e., ferulic acid-grafted chitosan in water.

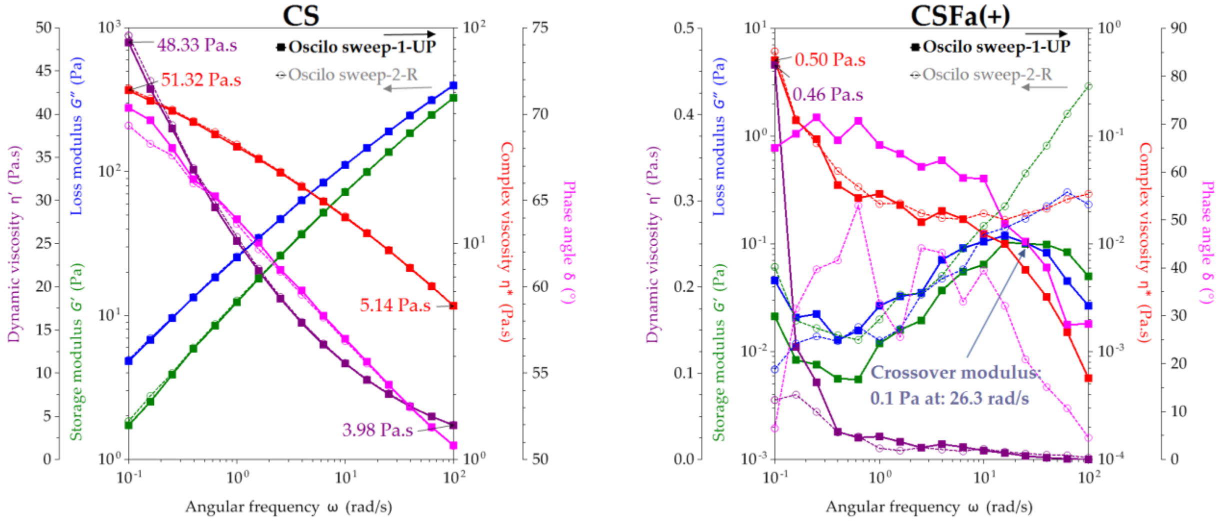

(**a**) (**b**)

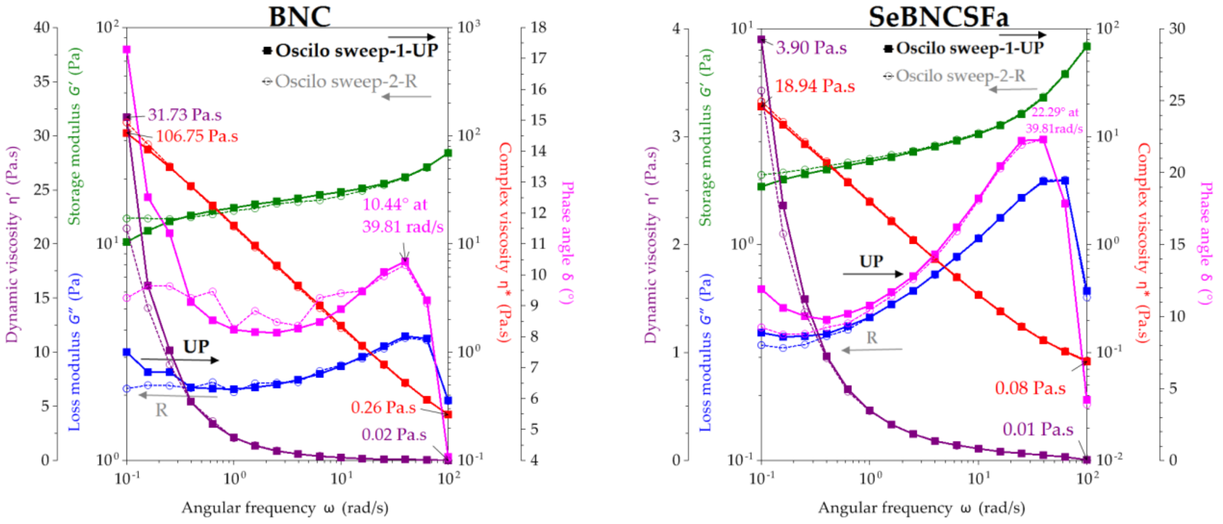

(c) (d)

Figure S13. Rheology experiments in oscillatory sweep mode: (**a**) CS; (**b**) CSFa(+); (**c**) BNC; (**d**) SeBNCSFa; CS: chitosan; CSFa(−): non-grafted chitosan; CSFa(+): ferulic acid-grafted chitosan; SeBNCSFa: hydrogel formulation based on ferulic acid-grafted chitosan and bacterial nanocellulose enriched with 2.5 μg/mL SeNPs from Kombucha fermentation.

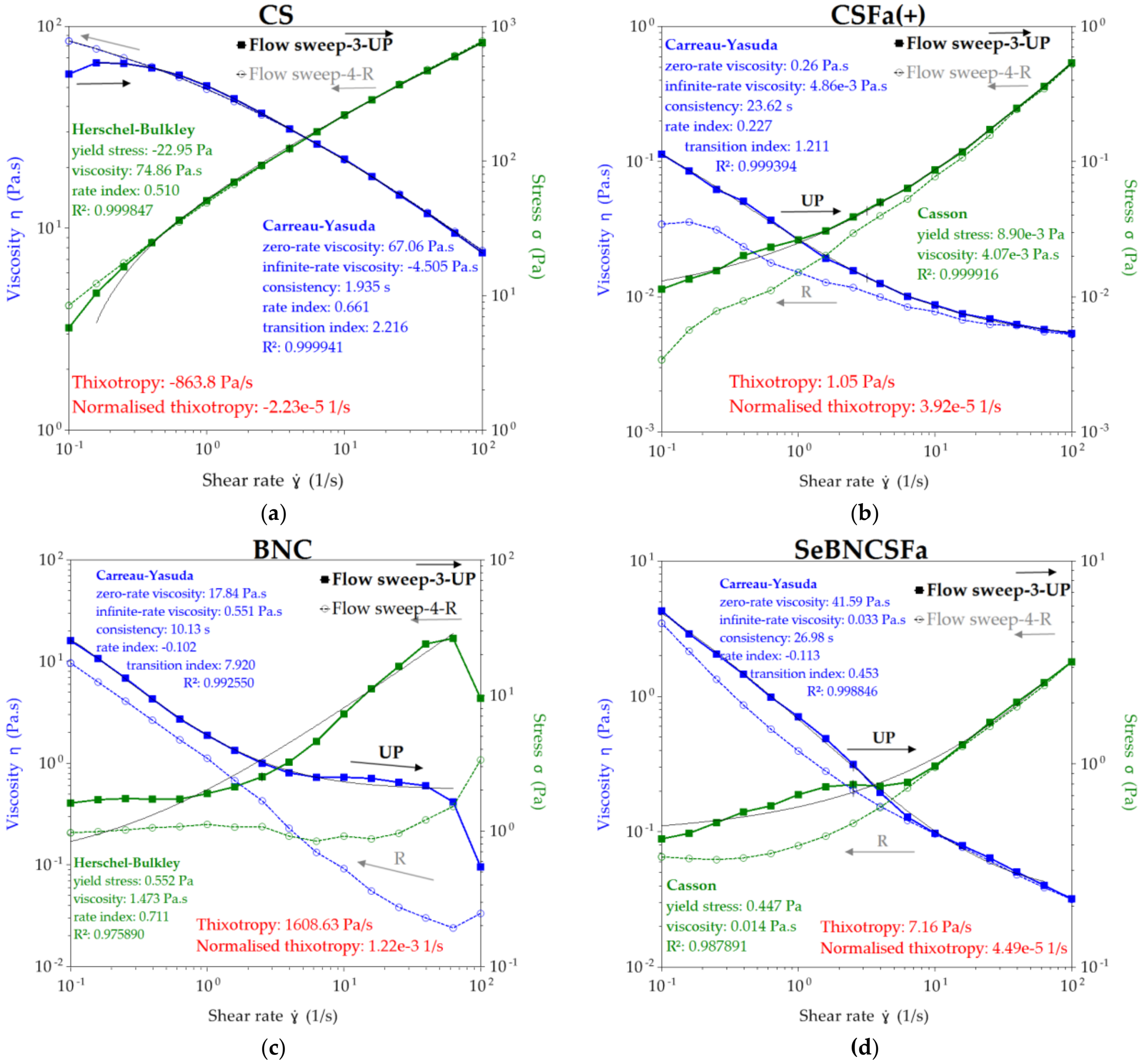

Figure S14. Rheology experiments in flow sweep mode: (**a**) CS; (**b**) CSFa(+); (**c**) BNC; (**d**) SeBNCSFa; CS: chitosan; CSFa(−): non-grafted chitosan; CSFa(+): ferulic acid-grafted chitosan; SeBNCSFa: hydrogel formulation based on ferulic acid-grafted chitosan and bacterial nanocellulose enriched with 2.5 μg/mL SeNPs from Kombucha fermentation.

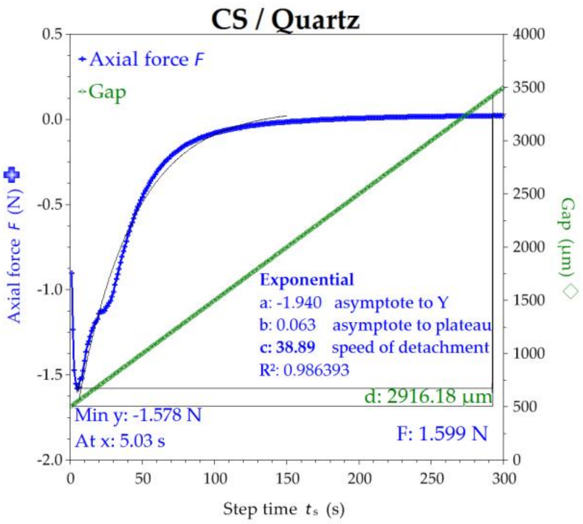

(**a**)

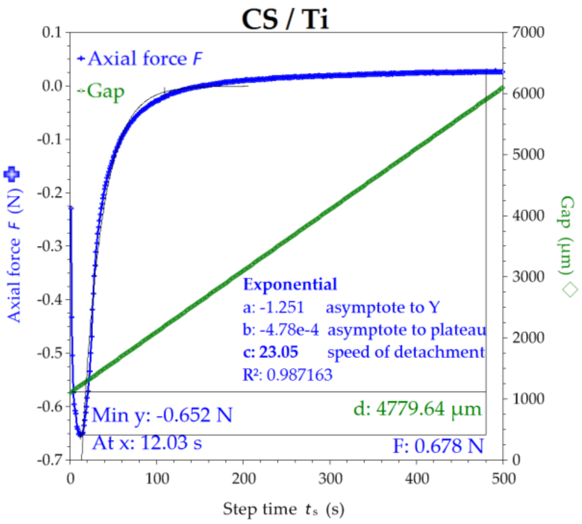

(**b**)

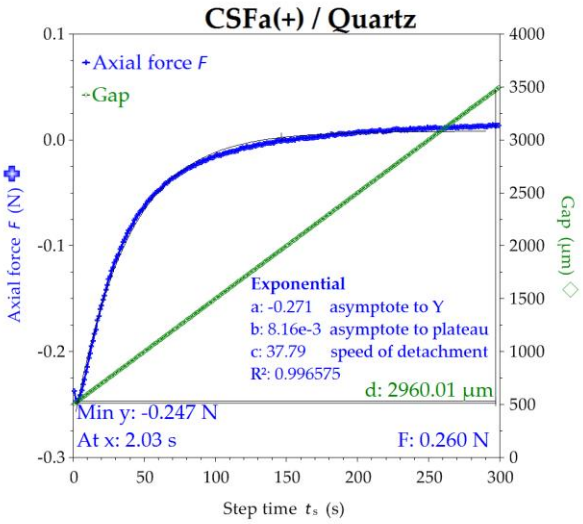

(**c**)

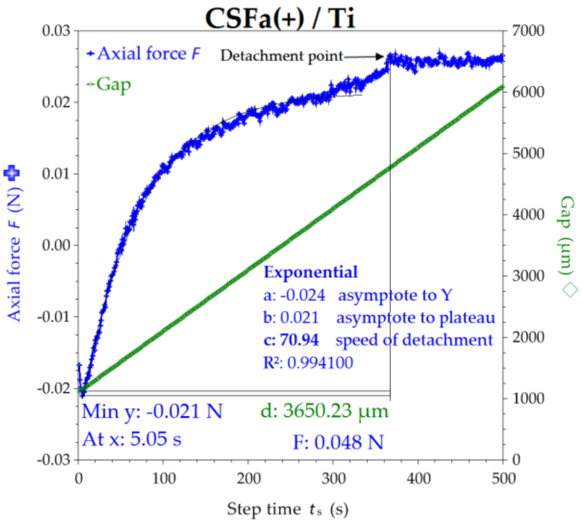

(**d**)

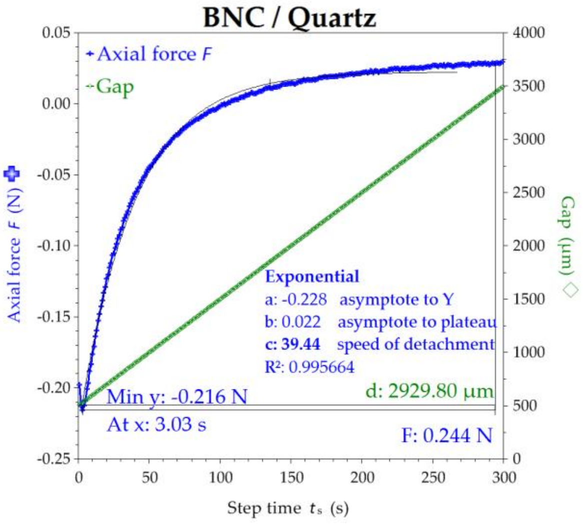

(**e**)

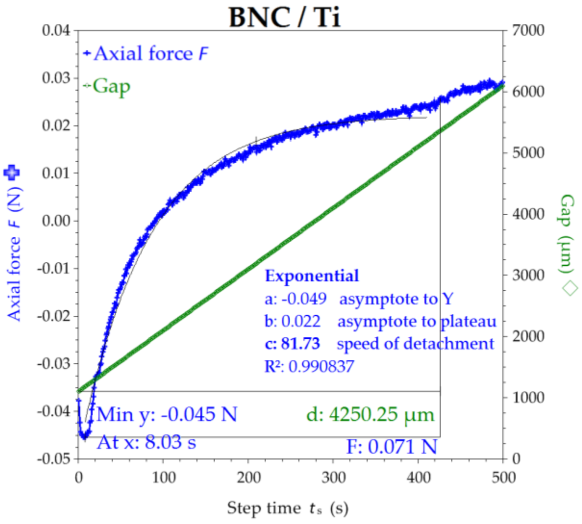

(**f**)

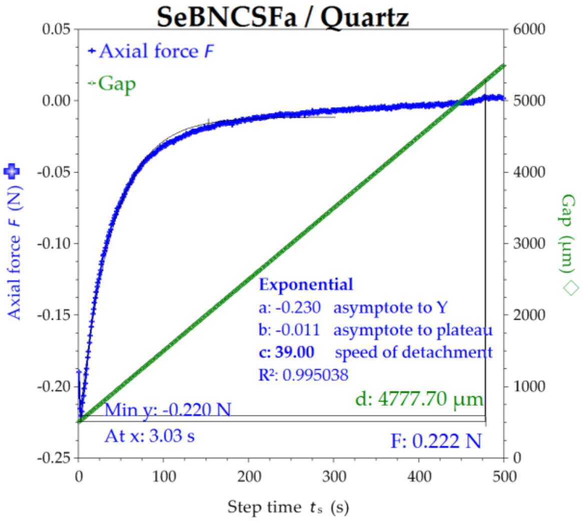

(**g**)

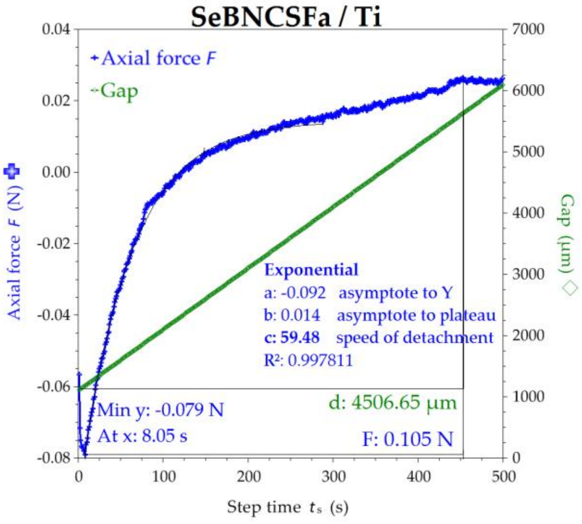

(**h**)

Figure S15. Rheology experiments in axial mode: (**a**) CS/Quartz; (**b**) CS/Ti; (**c**) CSFa(+)/Quartz; (**d**) CSFa(+)/Ti; (**e**) BNC/Quartz; (**f**) BNC/Ti; (**g**) SeBNCSFa/Quartz; (**h**) SeBNCSFa/Ti; CS: chitosan; CSFa(+): ferulic acid-grafted chitosan; BNC: bacterial nanocellulose; SeBNCSFa: hydrogel formulation based on ferulic acid-grafted chitosan and bacterial nanocellulose enriched with 2.5 µg/mL SeNPs from Kombucha fermentation.

Table S7. Screening of the bactericidal effect of SeBNCSFa.

**Strain**	***S. aureus***	***B. cereus***	***P. aeruginosa***	***E. coli***	***C. albicans***
**SeBNCSFa***	+	−	−	−	+

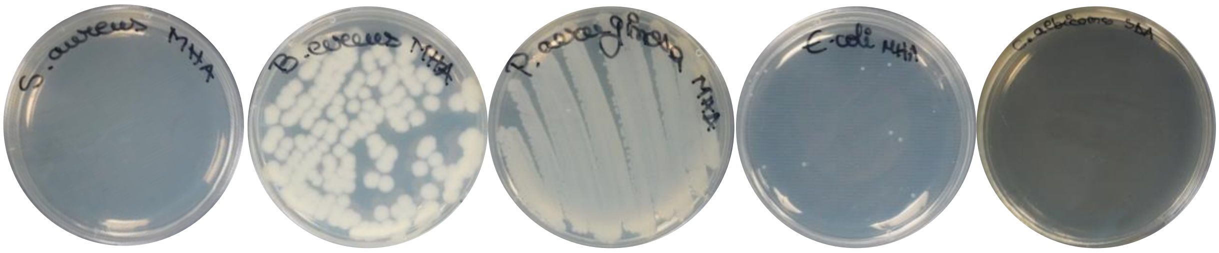

* SeBNCSFa: hydrogel formulation based on ferulic acid-grafted chitosan and bacterial nanocellulose enriched with 2.5 µg/mL selenium nanoparticles from Kombucha fermentation.

+ means bactericidal effect.

− means bacteriostatic effect.
